# A Case of Severe Hydroxychloroquine-Induced Retinal Toxicity in a Patient with Recent Onset of Renal Impairment: A Review of the Literature on the Use of Hydroxychloroquine in Renal Impairment

**DOI:** 10.1155/2012/182747

**Published:** 2012-12-09

**Authors:** Rajen Tailor, Ibrahim Elaraoud, Peter Good, Monique Hope-Ross, Robert A. H. Scott

**Affiliations:** Birmingham and Midland Eye Centre, City Hospital, Dudley Road, Birmingham B18 7QH, UK

## Abstract

We present a case of a 67-year-old female who presented with a twelve-month history of progressive blurred vision in both eyes. The patient was on hydroxychloroquine 200 mg twice a day for eight years for the treatment of scarring alopecia. Two years prior to presenting, the patient was found to have chronic kidney disease stage 3 secondary to hypertension. Examination revealed bilateral reduced visual acuities with attenuated arterioles and pigmentary changes on retinal assessment. Goldmann visual fields showed grossly constricted fields in both eyes. The patient was diagnosed with retinal toxicity secondary to hydroxychloroquine probably potentiated by renal impairment. Risk factors for retinal toxicity secondary to hydroxychloroquine can be broadly divided into dose-related and patient-related factors. Our patient developed severe retinal toxicity despite being on the recommended daily dose (400 mg per day). Although retinal toxicity at this dose has been documented, the development of renal impairment without dose adjustment or close monitoring of visual function is likely to have potentiated retinal toxicity. This case highlights the need to monitor renal function in patients on hydroxychloroquine. Should renal impairment develop, either the drug should be stopped or the dose reduced with close monitoring of visual function by an ophthalmologist.

## 1. Introduction

Hydroxychloroquine is commonly used for the treatment of systemic lupus erythematosus, rheumatoid arthritis, and other inflammatory and dermatologic diseases. Despite its common use, retinal toxicity from hydroxychloroquine has a low incidence [1]. However, retinal toxicity can have a devastating effect on vision, and even after cessation of this drug there may be little visual recovery and even progressive visual loss [2]. The risk of retinal toxicity increases with the duration of use (cumulative dose), age, preexisting retinal and macular disease, and renal or liver disease [3]. We report a case of severe retinal toxicity precipitated by renal impairment and review the literature on the use of hydroxychloroquine in renal impairment. 

## 2. Case Report

A 67-year-old female presented complaining of a twelve-month history of progressive bilateral blurred vision. Medical history included idiopathic scarring alopecia (patient started on hydroxychloroquine 200 mg BD 8 years previously) and chronic kidney disease (CKD) stage 3 (estimated-GFR >60), presumed secondary to hypertension (diagnosed 2 years previously). There was no family history of ocular disease.

Visual acuities were 6/24 on the right and 6/18 on the left. Retinal examination revealed bilaterally absent foveal reflexes, attenuated arterioles, and mild peripheral retinal pigmentary changes. Both discs appeared slightly pale.

**Figure 1:**
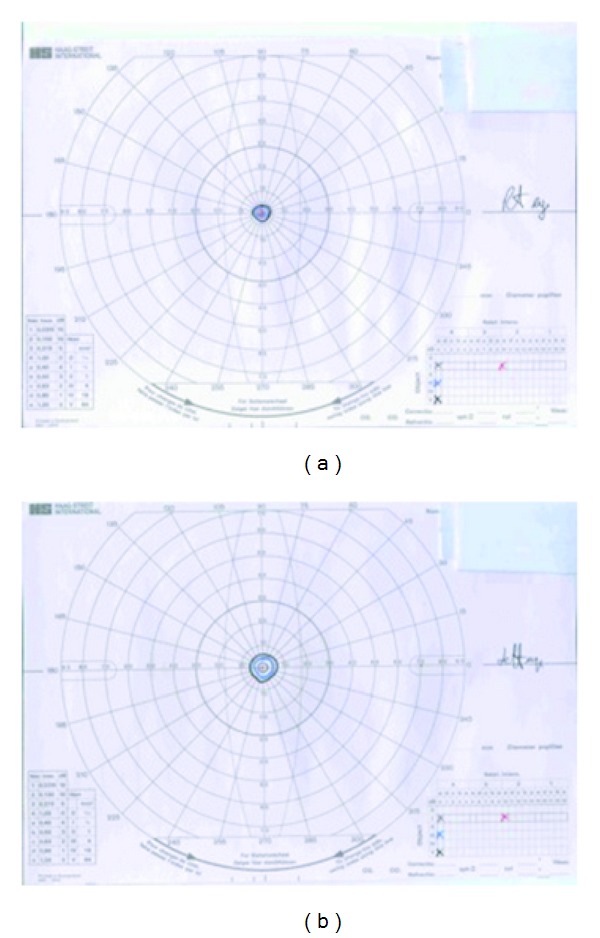
(a) Goldman Visual field of the right eye. Gross visual field constriction. (b) Goldman visual field of the left eye. Gross visual field constriction.

Goldman visual fields showed tunnel vision in both eyes with the field of vision reduced to 5 degrees on the right side (Figure 1(a)) and 10 degrees on the left side (Figure 1(b)). An electroretinogram showed bilateral absent or grossly reduced rod function and grossly reduced cone function. An OCT scan demonstrated the previously described “flying saucer” sign observed in patients with hydroxychloroquine-induced maculopathy [4].

Based on the above findings, a diagnosis of retinal toxicity secondary to hydroxychloroquine was made.

## 3. Discussion

Hydroxychloroquine is a quinolone. The highest tissue distribution is in the choroid and ciliary body of the eye. Hydroxychloroquine is partially metabolized, and 40–50% is excreted by the kidneys [5].

Ocular side effects can be divided broadly into corneal and retinal effects [6]. Corneal side effects include Hudson-Stahli line, verticillata, transient oedema, and decreased sensitivity. Retinal effects include retinal parafoveal granularity of the retinal pigment epithelium (RPE) with loss of the foveal light reflex (early in disease); bull's-eye appearance of the macula (late in the disease); vascular attenuation and peripheral fine granular pigmentary changes.

The risk of retinal toxicity secondary to hydroxychloroquine can be divided into dose-related and patient-related factors. For the former, it is the maximum daily dosage (>400 mg/day), the cumulative dose (>1000 g), and the duration of treatment (>5 years) that appear to be the most important determinants of the risk of toxicity [3]. Patient factors include obesity (if lean body weight is not used), small, thin elderly patients, and those with liver and renal impairment [3, 5].

The literature regarding the use of hydroxychloroquine in renal impairment is limited. The *Renal Drug Handbook* states that prolonged use in renal failure should be avoided. In patients with renal impairment, eye examinations should be carried out on a more frequent basis than annually [7].

 Dollery in The Renal Handbook [7] recommends that in renal impairment the dose of hydroxychloroquine for long-term use needs to be reduced according to the glomerular filtration rate (GFR) (Table 1).

Unfortunately, the manufacturers of hydroxychloroquine (plaquenil) have no information regarding its use in renal impairment.

The current Royal College of Ophthalmologist (UK) recommendations do not mention the use of renal impairment except to check baseline renal function [8]. A recent publication by the American Academy of Ophthalmology titled “Revised Recommendations on Screening for Chloroquine and Hydroxychloroquine Retinopathy,” [3] recommends annual screening in all patients after 5 years of hydroxychloroquine use. In addition, the paper recommends that physicians should advise patients to return ahead of scheduled screening if they develop new visual symptoms, new retinal disease, major weight loss, or liver or renal impairment [3]. The later must be emphasized to all patients on hydroxychloroquine treatment.

Our patient developed severe retinal toxicity and visual impairment despite being on the recommended daily dose (400 mg per day). Although retinal toxicity at this dose has been documented [9], the development of renal impairment without dose adjustment or close monitoring of visual function is likely to have potentiated retinal toxicity.

This case highlights the need to monitor renal function (and liver function) in patients on hydroxychloroquine. Should renal impairment develop, either the drug should be stopped or the dose reduced with close monitoring of visual function by an ophthalmologist.

## Figures and Tables

**Table 1:** Recommended daily dose of hydroxychloroquine according to the glomerular filtration rate (GFR) [7].

GFR (mL/min)	Maximum daily dose of hydroxychloroquine
20–50 mL/min	75 mg
10–20 mL/min	50 mg
Less than 10 mL/min	Contraindicated
